# Supplementary material for: Multifunctionalized Mesostructured Silica Nanoparticles Containing Mn2 Complex for Improved Catalase-Mimicking Activity in Water
Source: Nanomaterials (Basel). 2022 Mar 29;12(7):1136. doi: 10.3390/nano12071136 (PMC9000467; doi:10.3390/nano12071136)
Supplement: Supplementary file 1 [file nanomaterials-12-01136-s001.zip › nanomaterials-1634238-supplementary.pdf]

Supplementary Information

Multifunctionalized Mesostructured Silica Nanoparticles Containing Mn_2 Complex for Improved Catalase-Mimicking Activity in Water

Tristan Pelluau [1], Saad Sene [1], Beltzane Garcia-Cirera [2], Belen Albela [3,*], Laurent Bonneviot [3], Joulia Larionova [1,*] and Yannick Guari [1,*]

[1] ICGM, University Montpellier, CNRS, ENSCM, 34000 Montpellier, France; tristan.pelluau@umontpellier.fr (T.P.); saad.sene@umontpellier.fr (S.S.)
[2] Departament de Química Inorgànica i Orgànica, Universitat de Barcelona, 08028 Barcelona, Spain; beltzane.garcia@antares.qi.ub.edu
[3] Laboratoire de Chimie, ENS de Lyon, Université de Lyon, 69007 Lyon, France; laurent.bonneviot@ens-lyon.fr
* Correspondence: belen.albela@ens-lyon.fr (B.A.); joulia.larionova@umontpellier.fr (J.L.); yannick.guari@umontpellier.fr (Y.G.); Tel.: +33-472-728-856 (B.A.); +33-778-446-903 (Y.G.)

Table S1. Composition and molar mass of the nanoparticles determined by elemental analysis.

Name	Composition	M ($g.mol^{-1}$)
**MSN**	$(SiO_2)_1(CTA)_{0.005}$	61.4
**MSN-1a**	$(SiO_2)_1(CTA)_{0.005}(1,4\text{-pyr})_{0.008}$	63.2
**MSN-1b**	$(SiO_2)_1(CTA)_{0.006}(1,4\text{-pyr})_{0.047}$	72.2
**MSN-1c**	$(SiO2)_1(CTA)_{0.007}(1,4\text{-pyr})_{0.09}$	82.1
**MSN-2**	$(SiO_2)_1(CTA)_{0.005}$ $(SBS)_{0.03}$	70.8
**MSN-3a**	$(SiO_2)_1(CTA)_{0.005}(1,4\text{-pyr})_{0.017}(SBS)_{0.03}$	74.5
**MSN-3b**	$(SiO_2)_1(CTA)_{0.001}(1,4\text{-pyr})_{0.048}(SBS)_{0.028}$	79.7
**MSN-3c**	$(SiO2)_1(CTA)_{0.002}(1,4\text{-pyr})_{0.096}(SBS)_{0.027}$	90.1
**MSN-4a**	$(SiO_2)_1(CTA)_{0.001}(1,4\text{-pyr})_{0.096}(SBS)_{0.027}(Mn_2O)_{0.0096}$	98.4
**MSN-4b**	$(SiO_2)_1(CTA)_{0.001}(1,4\text{-pyr})_{0.096}(SBS)_{0.027}(Mn_2O)_{0.053}$	136.1

Table S2. ^{13}C chemical shift of the carbons from zwitterion (left side; **MSN-2**) and pyridine functions (right side; **MSN-1c**). The carbon count goes from the silicon atom to the end of the chain (see figure S13).

**MSN-2**	$\delta^{13}C$ (ppm)	**MSN-1c**	$\delta^{13}C$ (ppm)
1	17.6	1	12.3
2	17.6	2	12.3
3	59	3	12.3
4	59	4	148.2
5	59	5	123.7
6	17.6	6	154.7
7	49		

Table S3. Catalytic activity: Percentage of H_2O_2 decomposed and turnover number (TON).

		CH_3CN 1min	H_2O 1min	CH_3CN 5min	H_2O 5min	CH_3CN 10min	H_2O 10min	CH_3CN 20min	H_2O 20min
Free-Mn_2O	% H_2O_2	33	0.3	73	1	91	2	95	4
	TON	94	1	208	3	259	6	271	11
**MSN-4a**	% H_2O_2	44	63	89	100	100	100	100	100
	TON	125	179	253	285	285	285	285	285
**MSN-4b**	% H_2O_2	27	58	77	100	96	100	100	100
	TON	77	165	219	285	273	285	285	285

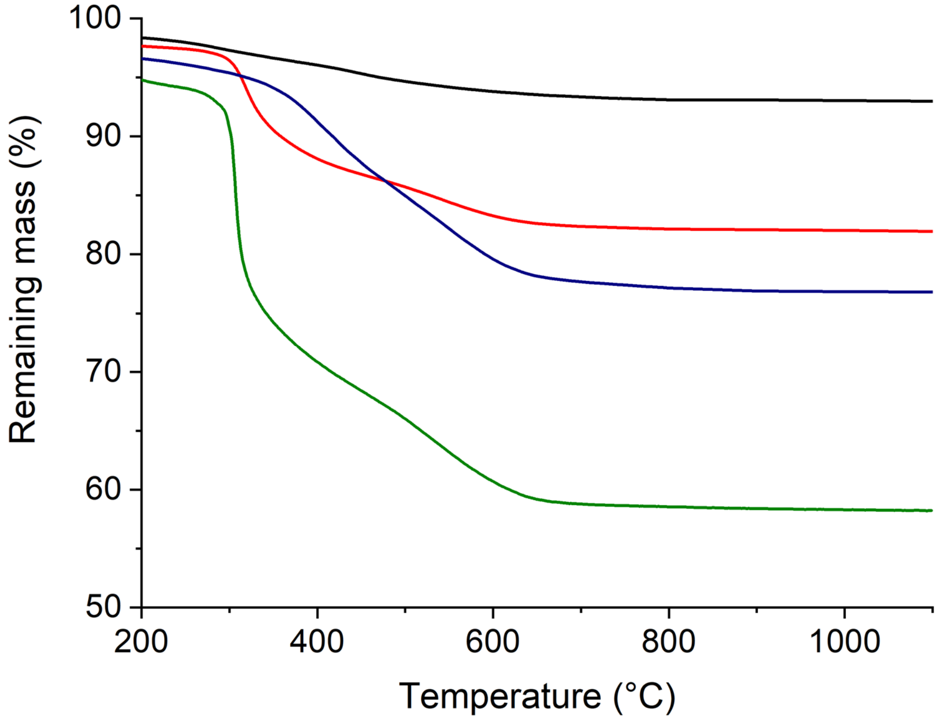

Figure S1. Thermogravimetric analysis (TGA) from 200°C to 1100°C at 5°C.min^{-1} under air of the nanoparticles with various amount of pyridine silane: **MSN** (black), **MSN-2 (**red), **MSN-1c** (blue), **MSN-3c** (green).

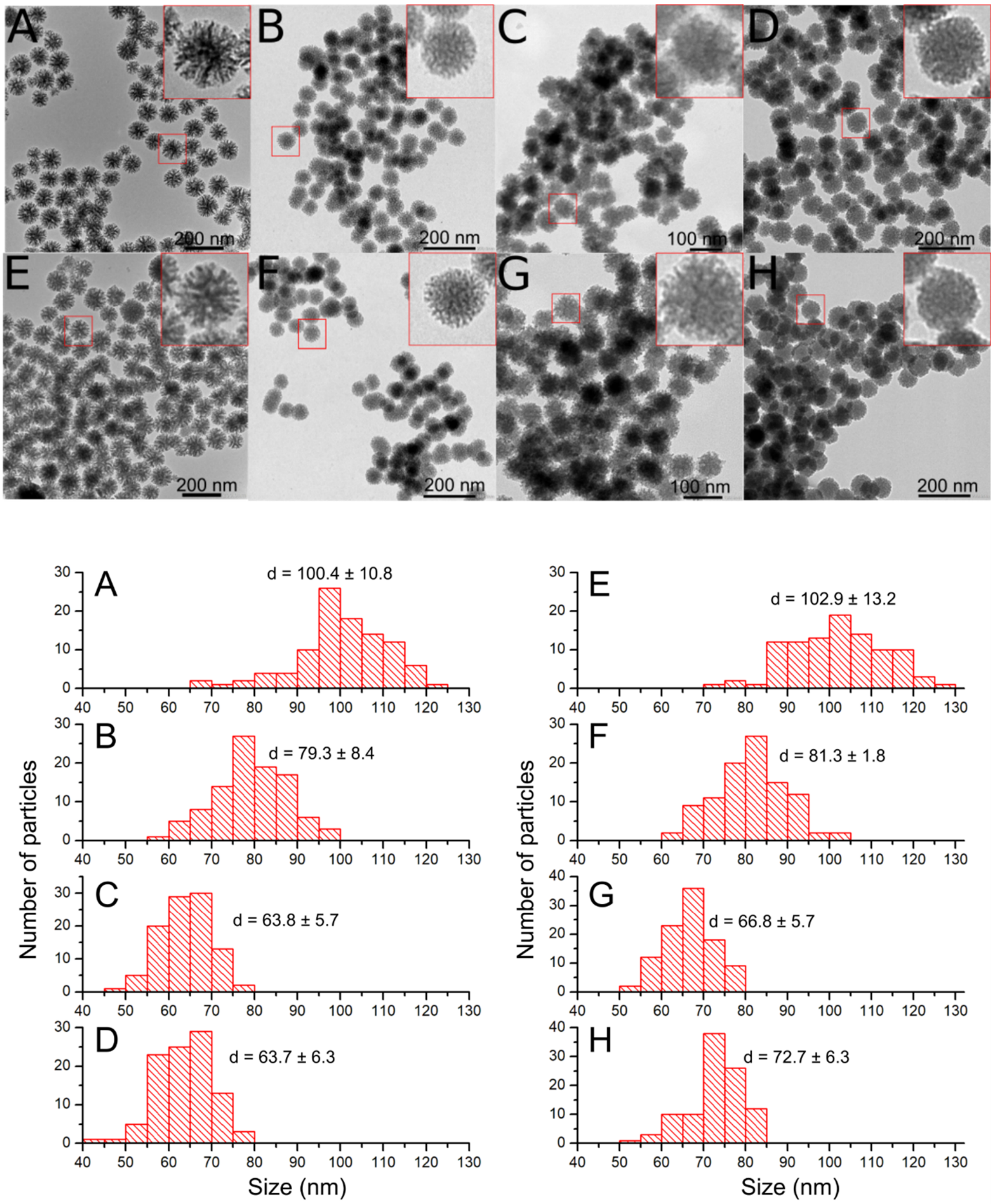

Figure S2. TEM images and size distribution of the nanoparticles with an increasing amount of pyridine with and without zwitterion grafted (**A**) **MSN**, (**B**) **MSN-**1a, (**C**) **MSN-1b**, (**D**) **MSN-1c**, (**E**) **MSN-2**, (**F**) **MSN-3a**, (**G**) **MSN-3b** and (**H**) **MSN-3c.**

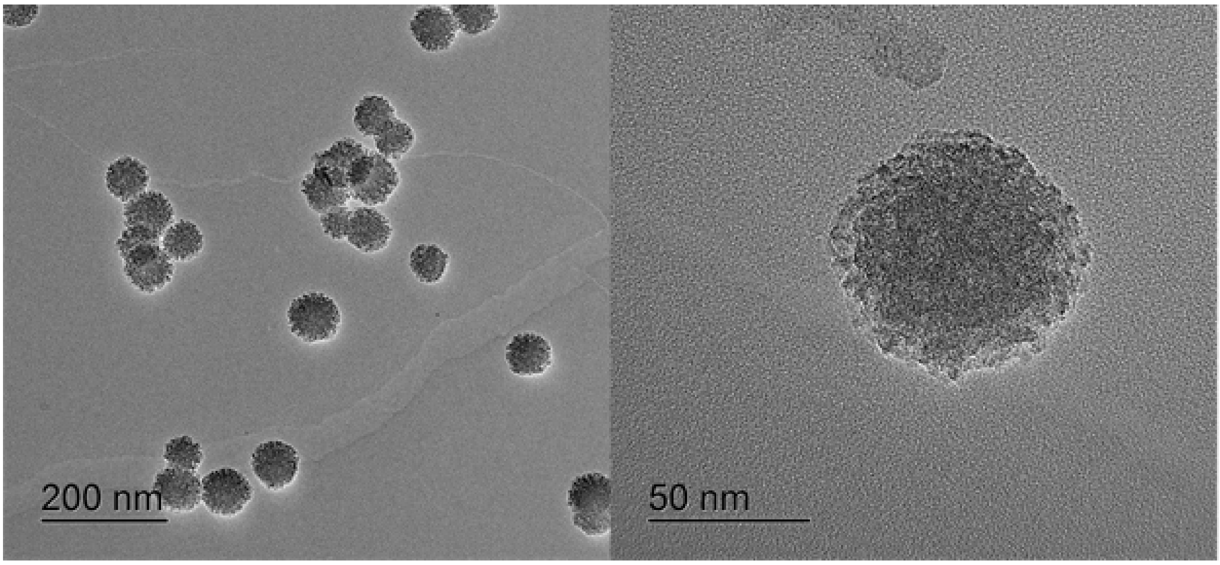

Figure S3. High Resolution TEM images of the complete nanoparticles **MSN-4b** with different magnifications.

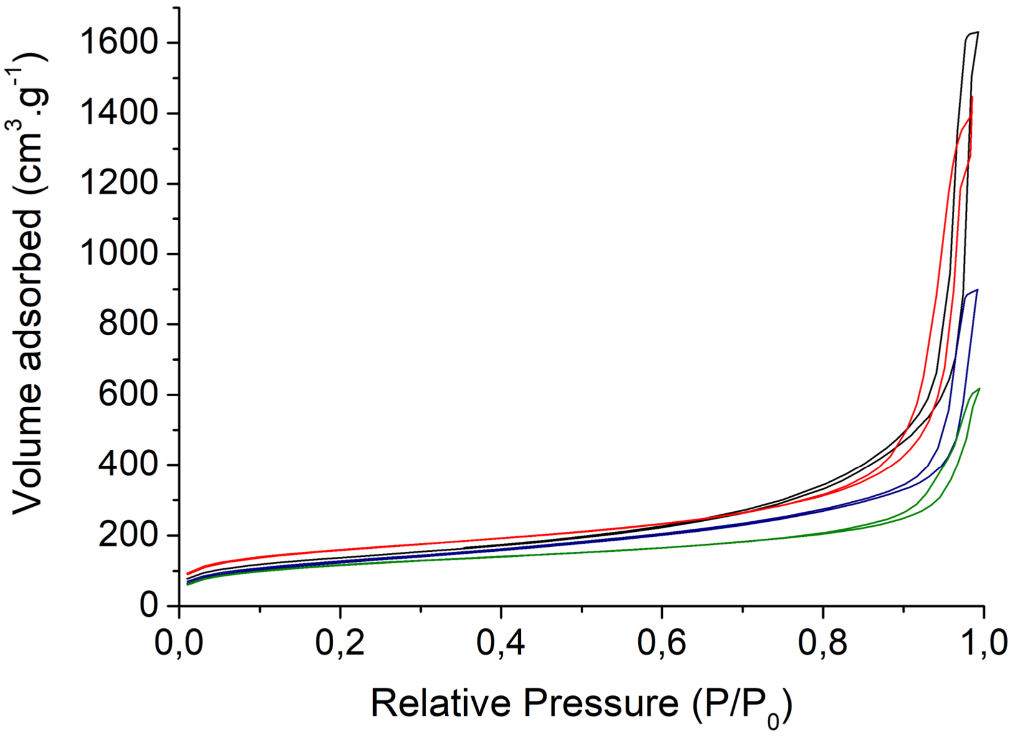

Figure S4. N_2 adsorption isotherms of silica nanoparticles with different amount of pyridine silane: **MSN** (black), **MSN-1a** (red), **MSN-1b** (blue) and **MSN-1c** (green).

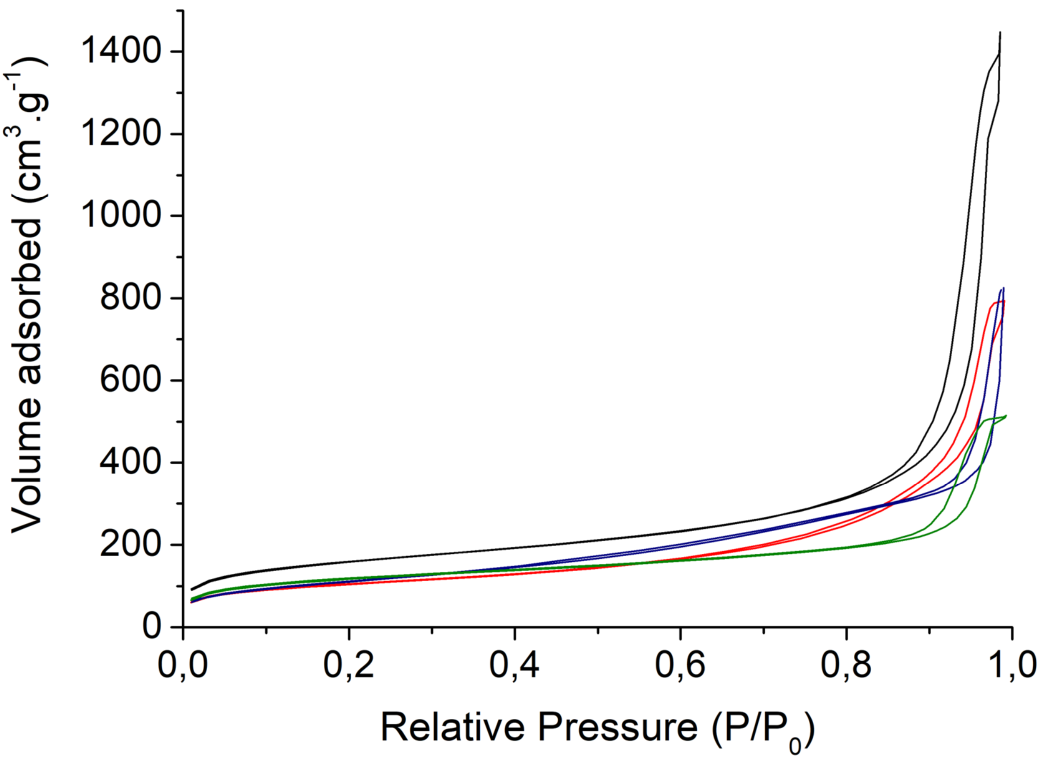

Figure S5. N_2 adsorption isotherms of silica nanoparticles with different amount of pyridine silane with zwitterion on the surface: **MSN-2** (black), **MSN-3a** (red), **MSN-3b** (blue) and **MSN-3c** (green).

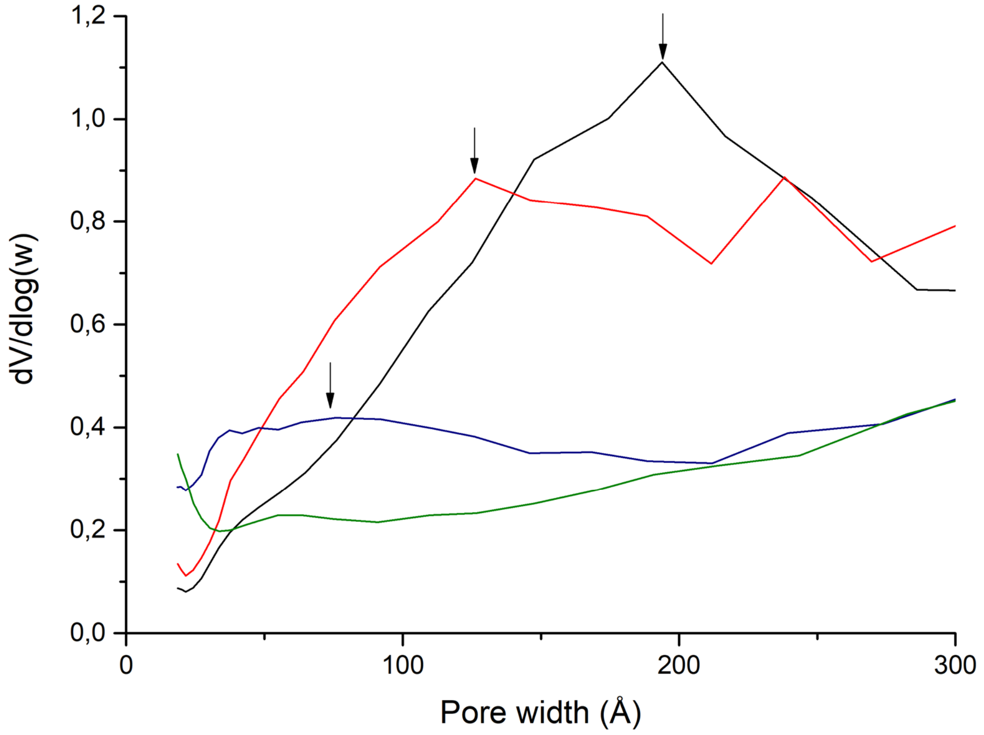

Figure S6. Pore size distribution curves calculated by BJH method of silica nanoparticles with different amount of pyridine silane: **MSN** (black), **MSN-1a** (red), **MSN-1b** (blue) and **MSN-1c** (green).

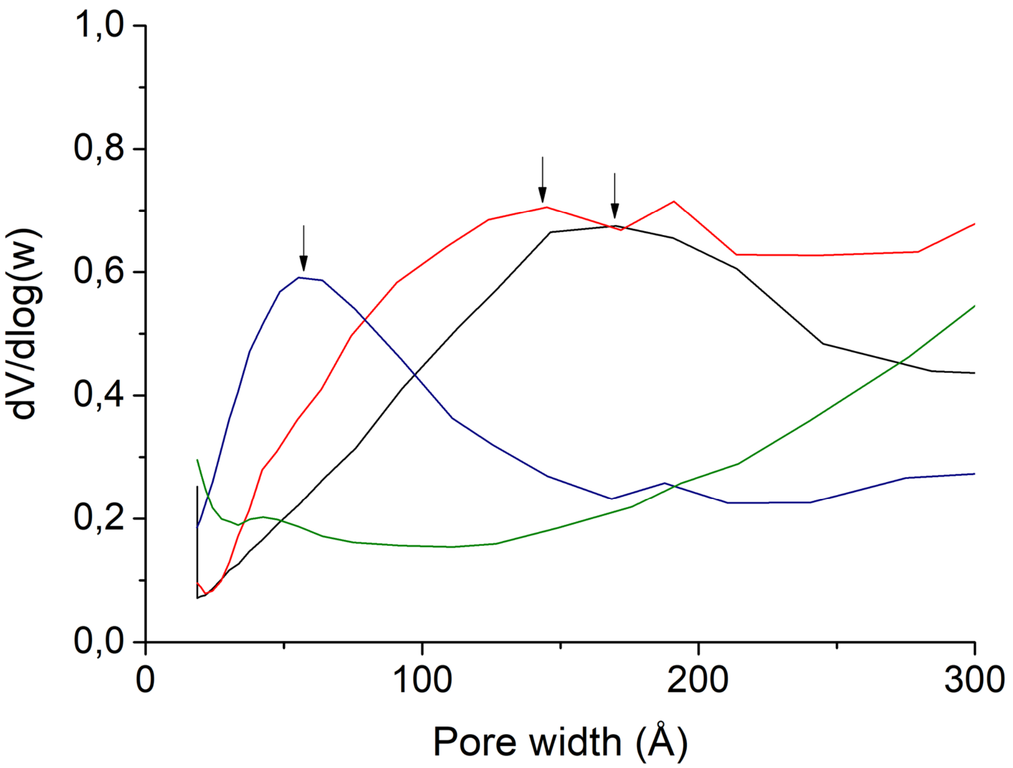

Figure S7. Pore size distribution curves calculated by BJH method of silica nanoparticles with different amount of pyridine silane with zwitterion on the surface: **MSN-2** (black), **MSN-3a** (red), **MSN-3b** (blue) and **MSN-3c** (green).

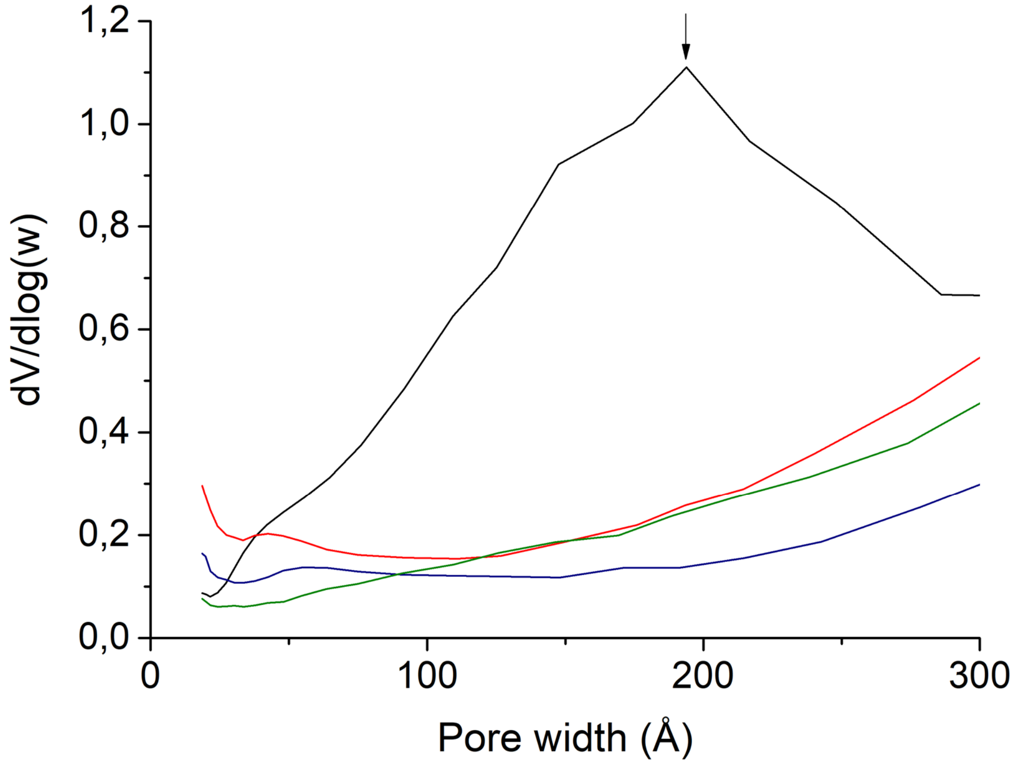

Figure S8. Pore size distribution curves calculated by BJH method of silica nanoparticles: **MSN** (black), **MSN-3c** (red), **MSN-4a** (blue) and **MSN-4b** (green).

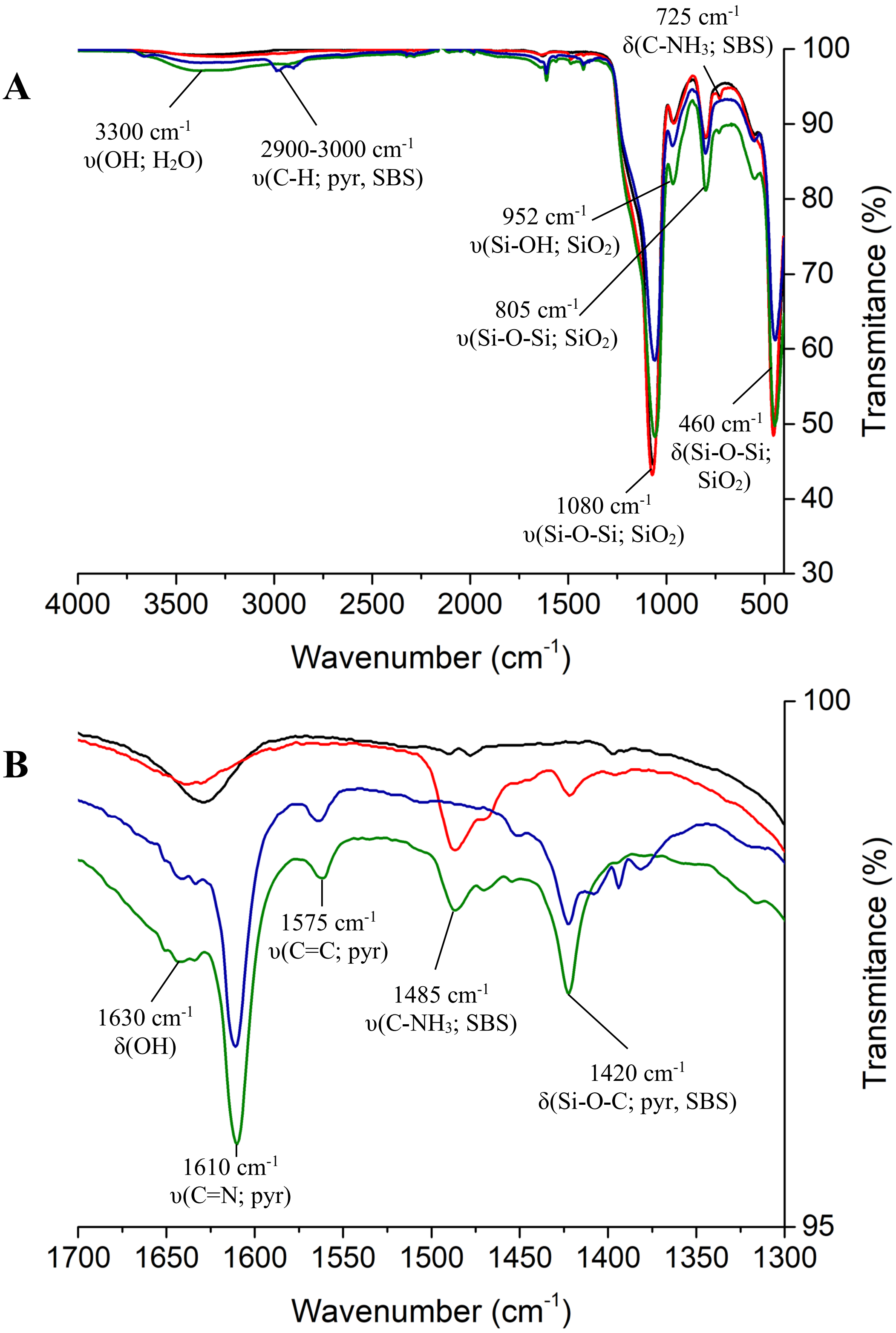

Figure S9. (**A**) Infrared spectra of **MSN** (dark), **MSN-2** (red), **MSN-1c** (blue), **MSN-3c** (green): (**B**) Magnification in the 1700-1300 cm^{-1} window.

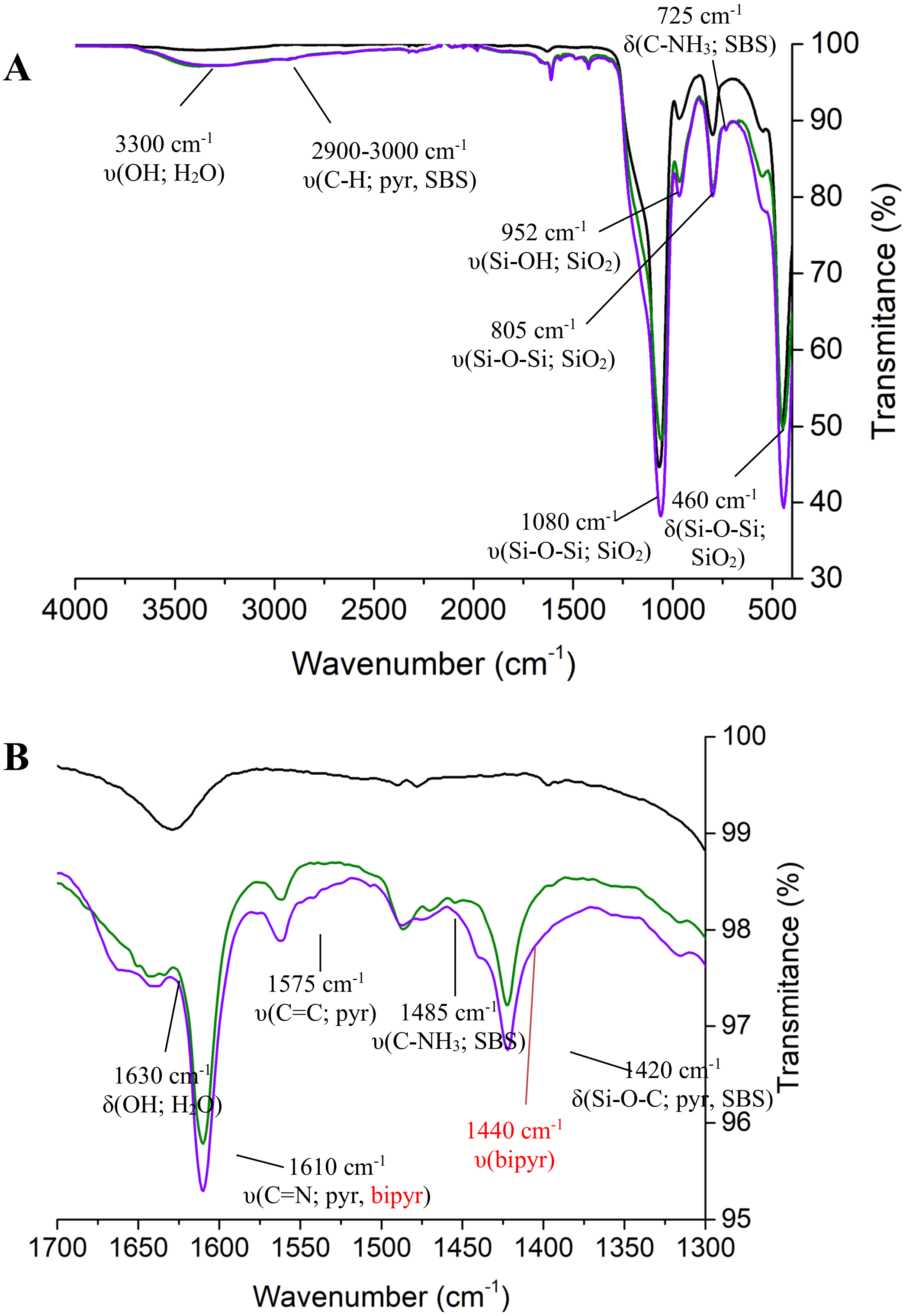

Figure S10. (**A**) Infrared spectra of **MSN** (dark), **MSN-3c** (green) and **MSN-4b** (purple); (**B**) Magnification in the 1700-1300 cm^{-1} window.

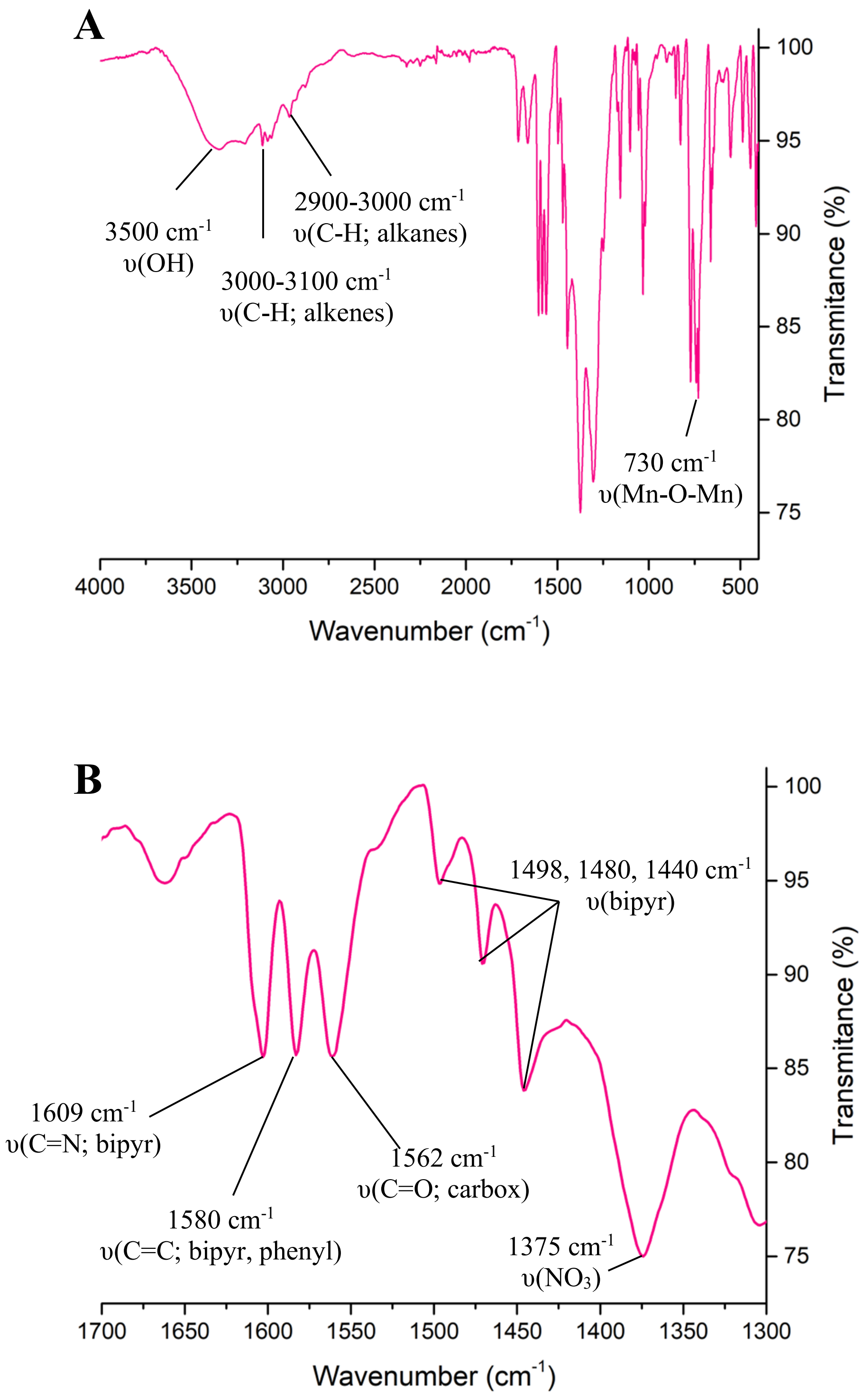

Figure S11. (**A**) Infrared spectra of [{Mn(bpy)(H_2O)}(μ-2-MeC_6H_4COO)$_2$(μ-O){Mn(bpy)(NO_3)}]NO_3 (**Mn_2O**); (**B**) Magnification in the 1700-1300 cm^{-1} window.

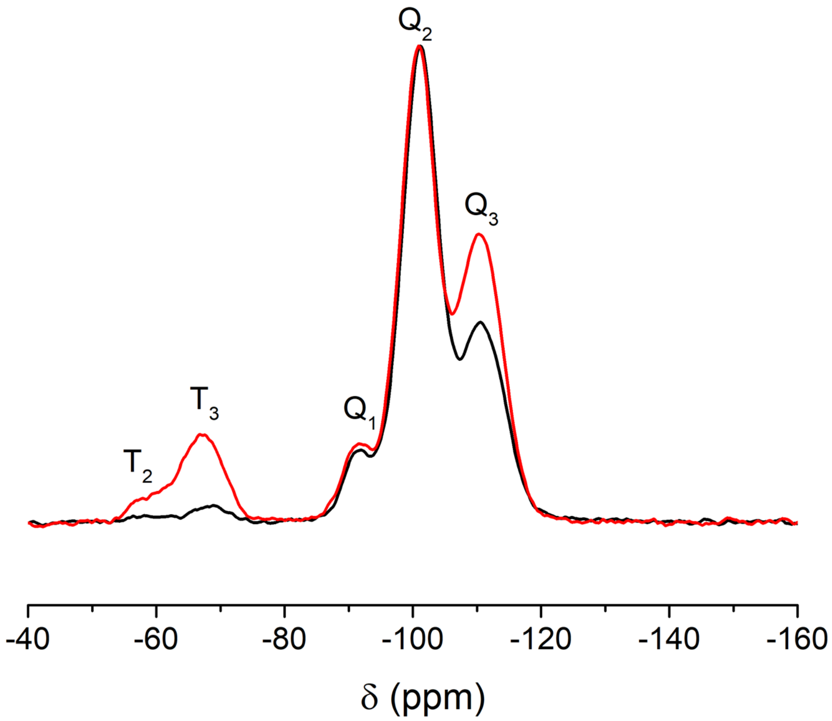

Figure S12. ^{129}Si CPMAS solid-state NMR of **MSN-1b** (dark) and **MSN-2** (red). CTA indicates the peaks from the remaining molecules of surfactant for both samples.

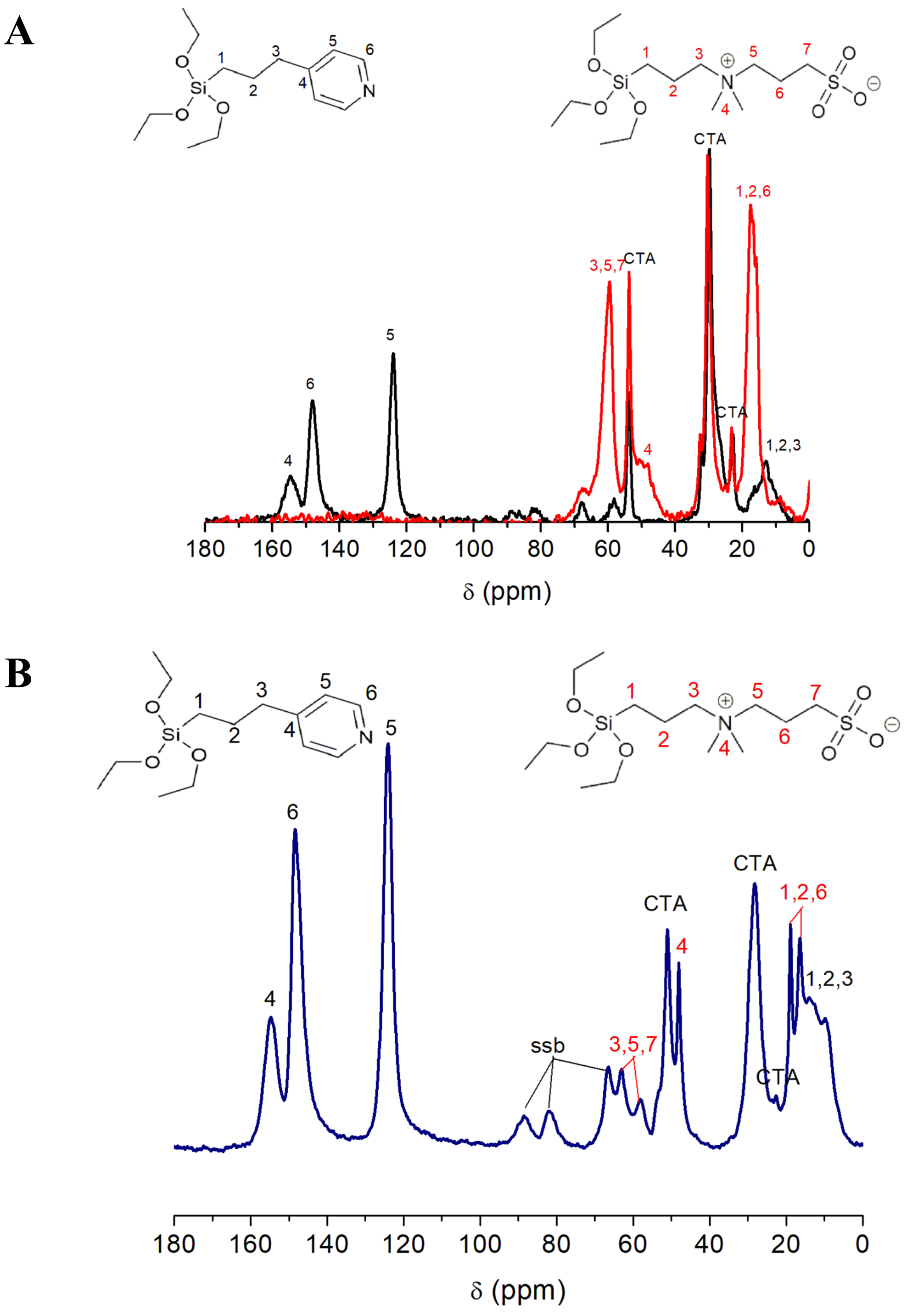

Figure S13. ^{113}C CPMAS solid-state NMR of (**A**): **MSN-1b** (dark) and **MSN-2** (red) and (**B**): **MSN-3c** (blue). CTA indicates the peaks from the remaining molecules of surfactant for all samples and ssb indicates the spinning sidebands from the pyridine peaks (4, 5, 6).

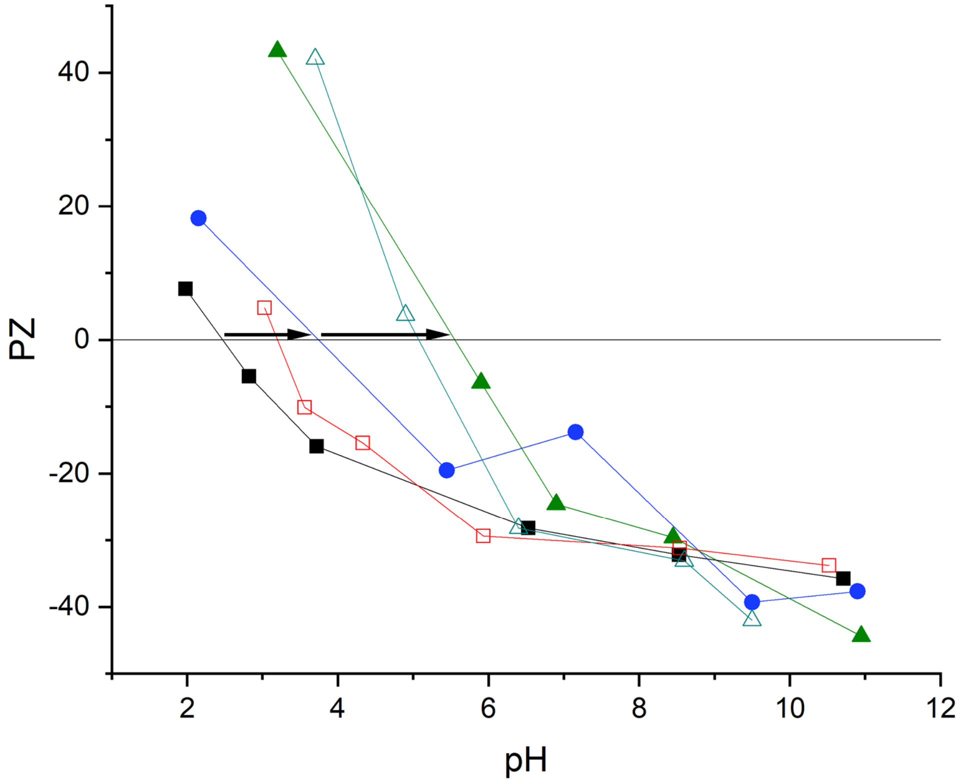

Figure S14. Zeta potential measurement of **MSN** (■), **MSN-2** (□), **MSN-1a** (●), **MSN-1c** (▲) and **MSN-3c** (△) as a function of pH.

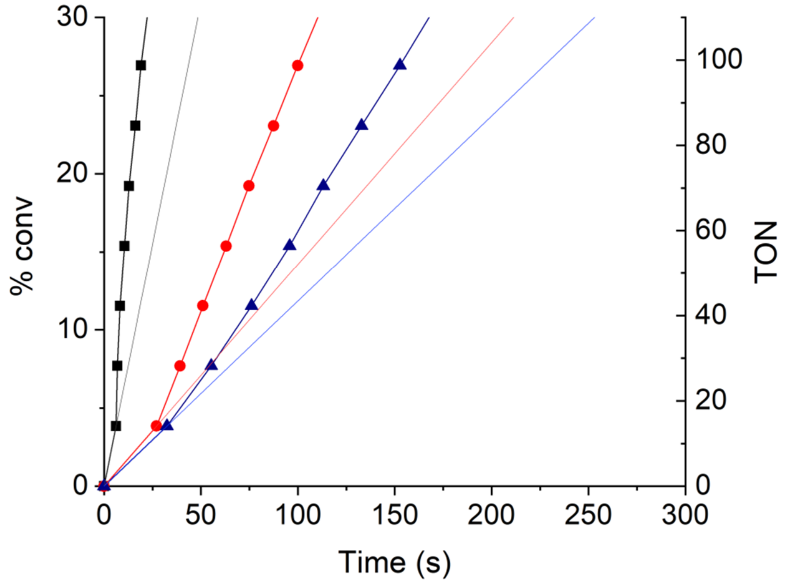

Figure S15. Percentage of H_2O_2 decomposed overtime by a suspension of **MSN-4a** in water and its Turnover Number (TON) for the 1st run (■), 2nd run (●) and 3rd run (▲).
